# An Exploratory Digital Board Game Approach to the Review and Reinforcement of Complex Medical Subjects Like Anatomical Education: Cross-sectional and Mixed Methods Study

**DOI:** 10.2196/33282

**Published:** 2022-01-10

**Authors:** Jun Wen Tan, Kian Bee Ng, Sreenivasulu Reddy Mogali

**Affiliations:** 1 Lee Kong Chian School of Medicine Nanyang Technological University Singapore

**Keywords:** serious games, board games, anatomy, flow

## Abstract

**Background:**

Serious games have the potential to resolve educational problems faced by medical students, such as insufficient rehearsal due to boredom and lack of motivation. However, serious games’ relatively novel concepts in science and many genres of games that are common in recreation remain underresearched in the literature. Board games are one such genre that, despite their potential, affordability, and flexibility, are rarely designed for medical students, and little is known about student perceptions of them and their compatibility with rehearsal.

**Objective:**

In this cross-sectional study, we sought to elicit, via an exploratory mixed methods approach, student perceptions of a digital serious board game specifically designed for the gamified rehearsal of complex medical subjects, with the chosen topic of anatomy.

**Methods:**

A digital serious board game, based on self-determination theory (SDT), was first designed and developed to facilitate the rehearsal of anatomy information. Students were then voluntarily recruited to partake in the intervention and were randomly split into three teams of 2 players per game session, after which they were administered the Flow Short Scale (FSS), which is a 13-item measure where items were rated on a 7-point Likert scale ranging from 1 (“not at all”) to 7 (“very much”). Students then participated in a focus group discussion to elicit their perceptions of the game. Findings from the FSS were subject to descriptive analysis, and the focus group discussion was subject to inductive thematic analysis.

**Results:**

A total of 12 undergraduate, second-year medical students from the Lee Kong Chian School of Medicine in Singapore participated in the study. FSS results indicated a moderate level of overall flow (mean score 4.94, SD 1.07) via the subdomains of fluency (mean score 4.77, SD 1.13) and absorption (mean score 5.21, SD 1.1). Students perceived the game as fun, enjoyable, engaging, and appropriate as a rehearsal tool that alleviated the monotony of traditional methods of rehearsal.

**Conclusions:**

Our digital board game–based rehearsal tool, when based on SDT, appeared to be suitable for gamified rehearsal in a fun and enjoyable environment due to its facilitation of intrinsic motivation in its players.

## Introduction

### Background

Modern medicine demands a skilled and adaptable workforce ready to take up an ever-expanding and increasingly complex knowledge base within constrained time frames [1,2]. Didactic methodologies no longer suffice, leading to modern student-centered approaches, such as flipped classrooms, problem-based learning, and team-based learning [3-6]. However, such methods focus on taking up novel information in place of retaining what has been learned. Very few consider how the affective and cognitive states of their learners might enhance, or be enhanced by, the methodologies in question.

Once a student graduates to clinical work, knowledge that is not immediately relevant is gradually lost without rehearsal [7]. At risk are complex cornerstone topics taught early in a student’s training, such as human anatomy and physiology. However, rehearsal itself may be impeded by numerous factors, such as disinterest when a student perceives lack of relevance of a complex but crucial topic with their intended field, or when the drive to rehearse is governed by contingent self-esteem (ie, the approval of others) [8].

This is a cause for concern due to the increasingly cross-disciplinary nature of medicine, where competency gaps may result in the preventable loss of life or limb. Woods et al [9] found that a mere 14% of final-year students were confident in anatomical knowledge. While students recalled two-thirds of unrehearsed knowledge at the conclusion of their preclinical years, this dropped by half after 2 years [10], further increasing the risk of preventable medical errors. The development of new methods of efficacious rehearsal are thus warranted.

Recreational video games enjoy near universal prevalence across cultures and age groups, and are receiving increasing attention from health care professionals due to their vast potential for training and therapy [11-13]. When games are purposefully designed for a nonrecreational—usually clinical or educational—application, they are termed as serious games [14,15]. Closely related is the concept of gamification, where a nongame application is enhanced with game-like elements for utilitarian purposes, such as increasing motivation and engagement [16].

When applied toward clinical training and education, serious games bear similarities to medical simulations in that they provide learners with opportunities for task training and practicing, provide active learning, aid in the solving of clinical problems, and afford experience in risk-free surroundings [17]. However, when designed well, they are also inspiring, engaging, and frequent conduits of an optimal, innately positive mental state characterized by psychological flow, motivation, and enjoyment [15,18,19]. These characteristics have seen them deployed for increasing psychomotor skills during laparoscopic surgery [20,21], teaching first-aid procedures in choking emergencies to nonexperts [22], and imparting history-taking content to medical students, among others [3,18,23].

### Board Games and Tabletop Games

The near-universal household penetration of digital games may result in physical play activities, such as board games and other tabletop games, being perceived as old-fashioned [24]. The digital aspects of such games include advantageous features, such as game analytics or, in the case of education, learning analytics [25,26]; ease of upscaling or mass deployment [27]; and ease of modification without the need to physically produce new materials [27].

Nonetheless, a sizable market for modern and newly recreated recreational tabletop games remains to this day, for such games are comparatively easier to design, their production is more economical, and they are natural conduits for social inclusivity [28]; thus, they remain viable avenues of exploratory research into novel game-based interventions. To this end, in the context of education, numerous board games have since been designed and trialed, including for antismoking education [29], nutrition education [30,31], infectious and parasitic diseases education [32-35], management of chronic diseases education [31,36-38], sexual health education [32,33,39], and anatomy education [40].

The socially inclusive and usually flexible nature of board games, a natural result of the need for multiple human players [28], also allows for the exploitation of game dynamics, defined as behaviors that players exhibit when interacting with the game and each other, for serious gains [24]. Game rules or features may be designed to elicit willful and positive interaction with otherwise boring learning materials, cooperation with other players to achieve a goal, or competition with said players to reinforce engagement in the activity [28]. Behaviors normally considered negative in the real world may also be leveraged for serious purposes; such dynamics include the use of trickery, deception, conspiracy, and even betrayal, assuming considerations for ethics have been fulfilled, for a more entertaining and engaging game activity [41-43].

Board and tabletop games have varied use in medical and clinical education. However, many of these games are basic in nature, bereft of complexity found in educational material for medical students, and are not usually focused on rehearsal. Notably, many lack clear theoretical foundations to their game’s design, a notable problem in the field, especially for early serious games [44,45], despite the dynamic and multifaceted nature of games qualifying them as complex interventions [46,47]. This risks unclear game mechanisms, conflicting frameworks of action, and questionable results wrought from unclear study designs [45,48,49].

### Study Aims

We proposed an exploratory study to gauge the feasibility of a purpose-built digital serious board game, primarily based on self-determination theory (SDT) as defined by Deci and Ryan [50,51], to enhance the rehearsal of anatomy among medical students, with the overall aim of determining their attitudes and perceptions toward such an intervention. Under this aim, the following research questions were investigated:

1. Is SDT a useful framework for the creation of digital board games?
2. What are students’ perceptions and attitudes toward the digital board game as a novel interventional tool for anatomy rehearsal?

## Methods

### Development of the Digital Board Game

**Figure 1:**
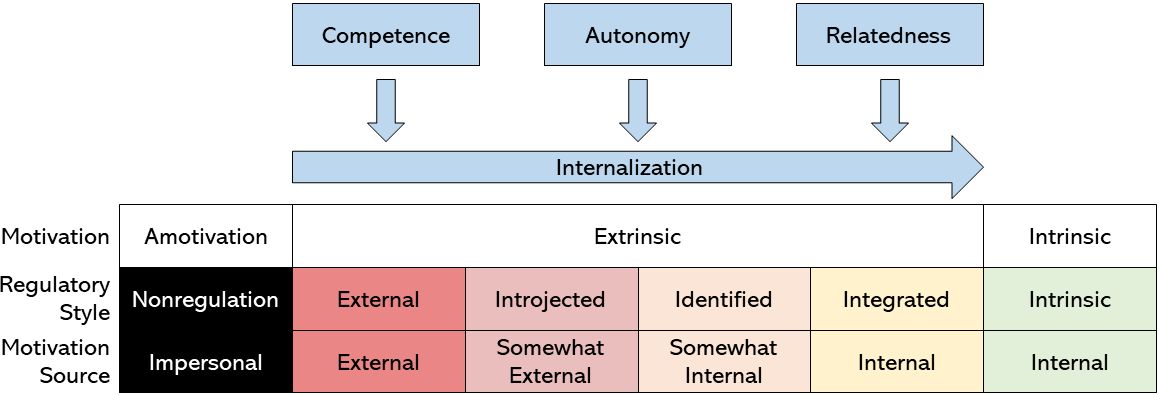
Self-determination theory as defined by, and adapted from, the works of Deci and Ryan [50,51]. The fulfilling of the three needs of competence, autonomy, and relatedness results in the promotion of internally regulated sources of motivation and, resultantly, an intrinsic desire to partake in the game activity.

SDT was chosen as the theoretical framework for the intervention due to its positing of a continuum between the wholly autonomous and wholly controlled behaviors resulting from factors that facilitate or undermine motivation [50,51]. Wholly autonomous behaviors are characterized by strong senses of choice and volition, are usually the result of intrinsic or internalized external motivators, and may be increased through fulfilling the psychological needs of competence, autonomy, and relatedness [50] (Figure 1). In contrast, the opposing end of the continuum describes wholly controlled behaviors usually regulated by compliance with greater powers, transactional rewards, or undesirable consequences should the behavior not be exhibited.

Of the needs required of an intrinsically motivating activity, *competence* is defined as a desire to master novel skills that may be met through the creation of challenging opportunities that individuals may partake in and progress through [51]. *Autonomy* is defined as the desire for control over one’s own destiny and behaviors; this desire may be met through activities that afford individuals the freedom to decide how to approach challenges, and how they wish to meet personal goals and exercise behaviors [51]. *Relatedness* is defined as the desire to develop and maintain close social relationships with others, be they as friends, partners, or groups, and this need may be met through activities that facilitate interpersonal connections, interaction, and teamwork in the context of competition [51].

SDT was thus used to guide the development of features and rule sets that would, as much as possible, allow for the internalization of the three needs and increase the amount of intrinsic motivation experienced by players, thereby keeping them engaged with the activity for as long as possible.

### Game Features, Rules, and Mechanics

The design of the game’s features and rule sets was primarily based on SDT to increase the amount of competence, autonomy, and relatedness felt by subjects playing the game.

To fulfill the need for competence, in-game educational materials were drawn from completed modules, and participants were permitted to look up answers from any source during play. To fulfill the need for autonomy, game rules were intentionally kept simple, minimally restrictive, and primarily enforced around the rehearsal component. To fulfill the need for relatedness, the game was played in cooperative two-person teams.

**Figure 2:**
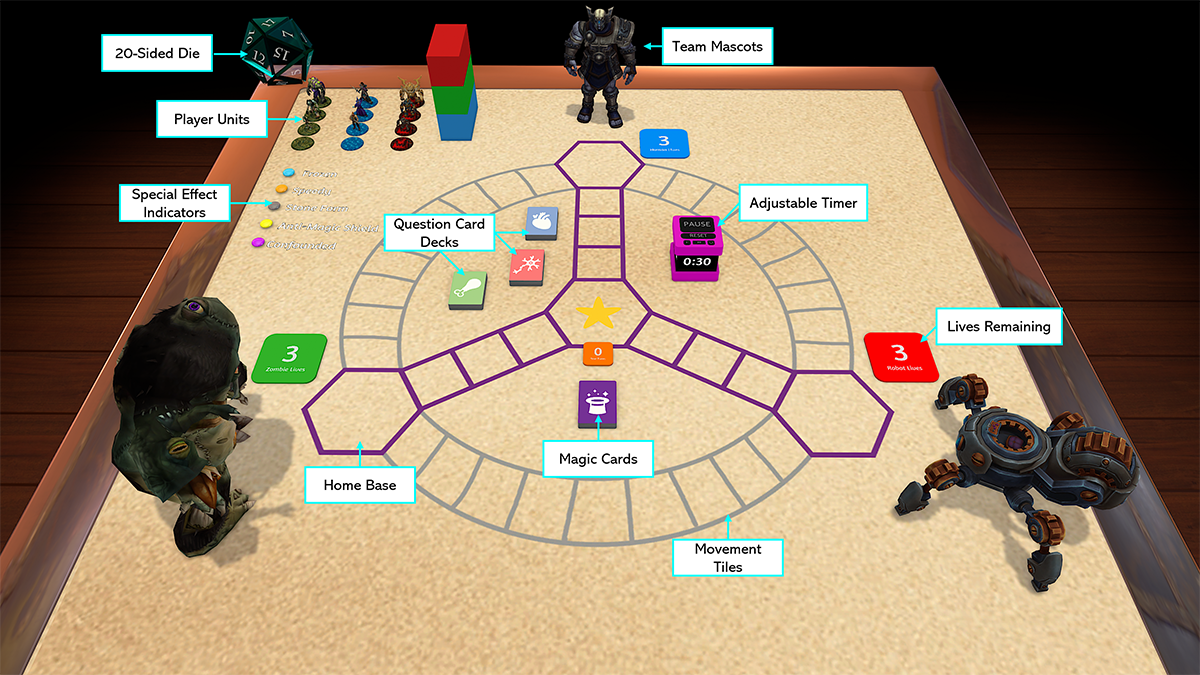
Overview of the game board and all elements therein. Only one copy of each team’s unit is featured, due to a duplicate function that allows for easy "copying and pasting" of any item on the board.

**Figure 3:**
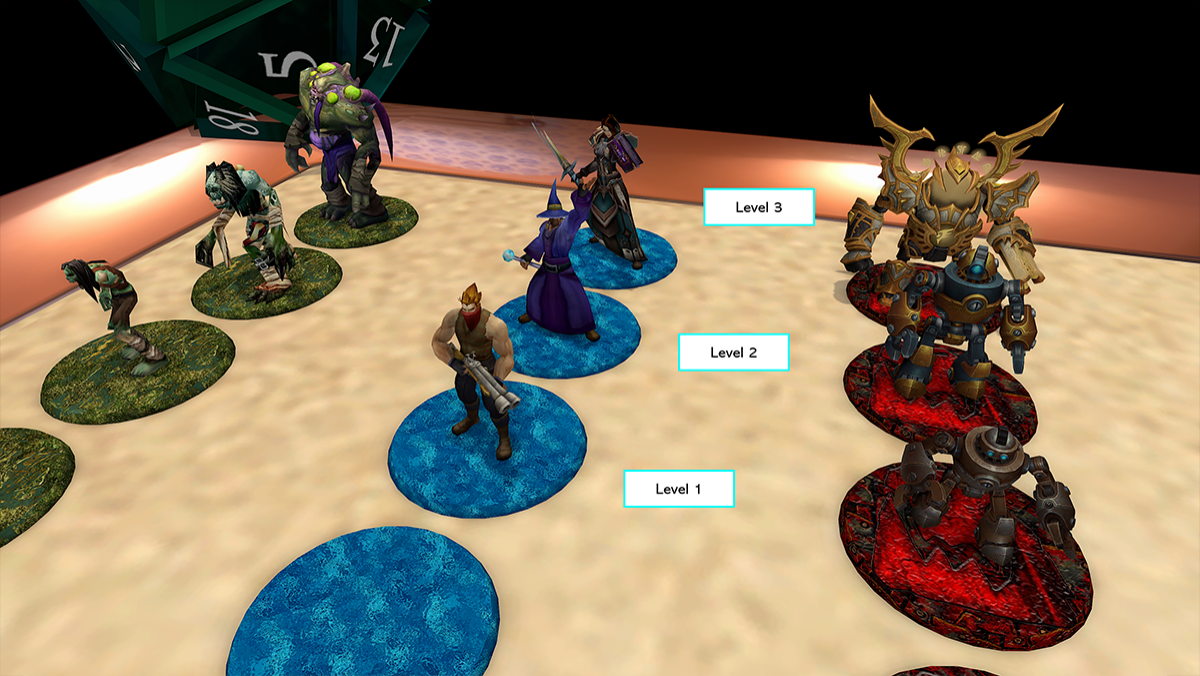
Close-ups of each team’s functionally identical but cosmetically distinct units. All units were equipped with colored bases for ease of locating when the camera was raised high above the game board.

The game’s final iteration was a turn-based free-for-all competition between three teams tasked to eliminate the other two (Figures 2 and 3). Each team may accomplish this through the generation of cosmetically distinct but functionally identical units, and then navigating said units into opposing bases to remove one life from an opponent. The cosmetic differences between teams, represented by team mascots, correspond to the three key topics of anatomy represented in the game. Internal organ systems are represented by humans, the musculoskeletal system is represented by zombies, and the nervous system is represented by robotic units.

**Figure 4:**
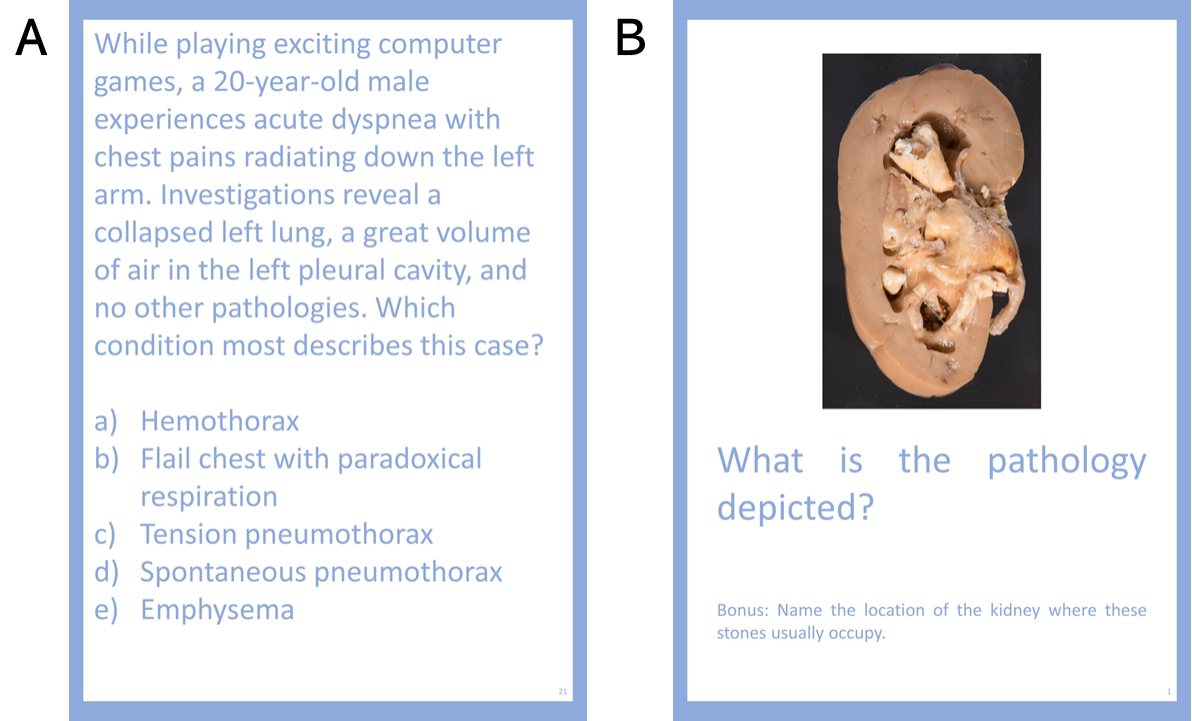
Question cards are shown, which are comprised two types of questions: (A) multiple-choice questions and (B) open-ended questions requiring students to specify short answers.

Unit generation may only be accomplished through interaction with the question card rehearsal activity. Decks are represented by a heart for internal organs, a nerve for the nervous system, and a chicken drumstick for the musculoskeletal system. At the start of their turns, teams may elect to draw a random question card from one of the three decks for either a multiple-choice question testing application of knowledge or an open-ended image card testing identification of anatomically relevant structures (Figure 4). Teams are permitted to look up answers to questions, but they must answer questions within a fixed time (default 30 seconds) to maintain a sense of urgency and prevent other teams from growing bored.

Upon successful answering of a question, players are given a level-1 unit that they may either opt to deploy to the board immediately or retain for upgrading to a level-2 or -3 unit, subject to answering more question cards on their next turn. Units of different levels have health points, and they deal to other units a value of damage equal to their level (ie, a level-3 unit has 3 health points and deals 3 points of damage); units that receive damage are reduced in level if they do not lose all their health points in the confrontation (ie, a level-3 unit that receives 1 point of damage from a level-1 unit is downgraded to a level-2 unit). This feature serves to reduce the life expectancy of deployed units and indirectly incentivizes partaking in the rehearsal feature.

To further reduce life expectancy of deployed units, the board’s tiles were designed such that players may opt to take two paths to their opponents. While grey tiles indicate a longer but safer path (nine tiles), purple tiles are shorter (seven tiles) and include an additional incentive of a “star tile” at the central intersection. Teams who successfully control the star tile for three turns in a row are allowed to draw a magic card that allows them to cast either beneficial effects on themselves or detrimental effects on their opponents.

To ensure no one team, either through luck or unusual competence, may dominate the board for too long and irreversibly damage morale in their opponents, a rule was included that awards any team that loses a life one magic card for immediate use.

### Participants

Subjects were recruited from the group of second-year medical students undertaking their Bachelor of Medicine, Bachelor of Surgery (MBBS) degree at the Lee Kong Chian School of Medicine, Nanyang Technological University in Singapore. Second-year medical students were selected due to their having completed all systems-based anatomy modules required for their clinical years. Information about the study and links for registering one’s interest were disseminated to prospective students via email advertisements, as well as advertisements during the break periods of student lessons. Students were repeatedly informed that participation was voluntary and had no bearing on their educational journey or course credits. Students diagnosed with, or suspected to suffer from, epilepsy, vision problems (severe myopia, photophobia, reduced visual acuity, eye strain, severe dry eyes, etc), significant psychosocial problems, or any other characteristics that may put them at risk as a result of study participation were excluded from the study.

All student participants were sent, and instructed to read, the study information sheets upon registration. All were rebriefed on the study’s procedures on the actual day of the study, and a second copy of the study information sheet was presented to them prior to the collection of consent. Ethical approval was obtained from the Nanyang Technological University Institutional Review Board (IRB-2021-01-038-01).

### Evaluation of the Intervention

**Figure 5:**
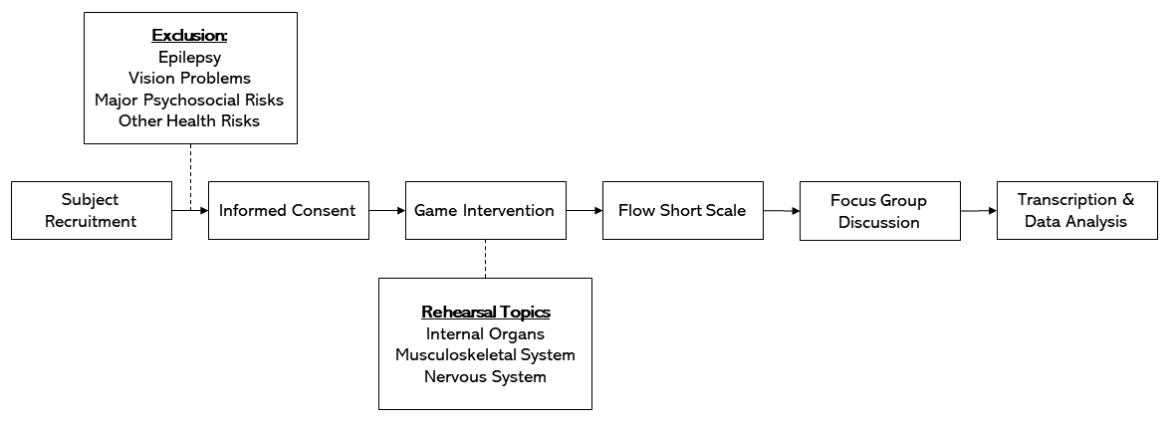
Overview of the mixed methods study design, wherein collection of quantitative data comprised the Flow Short Scale and qualitative data was comprised of transcriptions from the focus group discussions.

This cross-sectional study employed a mixed methods exploratory approach (see Figure 5 for an overview) conducted in two sessions to evaluate the digital game for anatomy rehearsal. For each session, 6 voluntary subjects—the maximum allowed by the board game—were randomly sorted into three teams of 2 required to play the game. The game activity lasted approximately one hour and was comprised of a blend of play and rehearsal; the latter featured material drawn from the entirety of the students’ anatomy education split into internal organs, the musculoskeletal system, and the nervous system. Students remained blinded to the topics until commencement of the activity. Rehearsal materials were presented as question cards containing either multiple-choice or open-ended image questions, and students were, by default, given 30 seconds to answer each question. The game was conducted as per the rules described in the above sections. During all rounds of play, a member of the study team (JWT) facilitated the group to ensure compliance to the basic rules and checked all proposed answers against an answer sheet. Participants from the first session were instructed not to reveal the study details, methods, and procedures to other students, in order to maintain the integrity of the experiment.

### Flow Short Scale

After the intervention, the Flow Short Scale (FSS) was administered; this scale is a 13-item measure, with items rated on a 7-point Likert scale ranging from 1 (“not at all”) to 7 (“very much”). The FSS has demonstrated construct validity [52] and good psychometric properties (α=.90), and it measures a stable three-factor structure comprising fluency of performance, absorption by activity, and perceived importance or outcome importance of the activity [53]. The scale is most frequently used as a retrospective measure of flow experienced during an immediately preceding activity. To eliminate the risk of fomites, the FSS was digitized and transmitted to subjects via a QR code to university-secured Google Forms.

### Focus Group Discussion

Following the FSS and before participating in focus groups, participants were reminded that the focus group discussions would be recorded for transcription by a third-party transcription company. All focus group discussions were assigned facilitators who had previously been familiarized with the game intervention, who were not present during gameplay, and who themselves had no expertise with anatomy. Guiding questions comprised the following:

1. Was there anything you found memorable when playing this game?
2. How would you change this game to make it more helpful?
3. How could we have made the game more fun for you?
4. When did you feel yourself entering a state of flow and what were you doing just before?

### Data Analysis

Quantitative data were comprised solely of the FSS results, with the first 10 items, measuring the dimension of flow, tallied and the mean derived as an indicator of how much flow was experienced. The last three items, measuring perceived importance, were similarly averaged.

The transcribed focus group discussions were subject to inductive thematic analysis in accordance with guidelines established by Braun and Clarke [54], due to the exploratory nature of the study and the lack of existing theoretical models describing user engagement in educational board games. Transcriptions were analyzed by two authors (JWT and SRM) who have extensive experience with qualitative research. While both have had prior experience playing board games, none actively or regularly play in the present day. Prior to coding, both authors familiarized themselves with the data by reading through the transcripts. A selective coding process was administered given the study’s exploratory nature and the need to elicit the perceived relevance and value of the game for student rehearsal, that is, factors that facilitated the recall of information, personal enjoyment, and elements, if any, that were perceived to assist in increasing the efficacy of the intervention [54]. Both authors coded independently with no input from the other until completion. Codes were then compared, and differences were discussed until a resolution was reached.

## Results

### Overview

A total of 12 subjects participated in the study; this included 7 males (58%) and 5 females (42%), and subjects were aged between 20 and 23 years (mean 20.91, SD 0.99). Sessions comprised 4 males and 2 females in the first group, and 3 males and 3 females in the second group.

### Flow Short Scale

The results of the FSS, whose items were rated on a 7-point Likert scale ranging from 1 (“not at all”) to 7 (“very much”), indicated a moderate degree of overall flow resulting from moderate levels of fluency of performance and absorption by activity subdomains. The perceived importance of the activity, independent from flow, was low (Table 1).

**Table 1:** Results of the Flow Short Scale (FSS) indicating moderate levels of flow via the subdomains of fluency and absorption, and a low degree of perceived importance.

Subdomain	Female participants (n=5), FSS score^a^			Male participants (n=7), FSS score			Total participants (N=12), FSS score	
	Range	Mean (SD)	Range		Mean (SD)	Range		Mean (SD)
Overall flow	3.94-5.96	5.00 (0.81)	2.46-6.22		4.90 (1.29)	2.46-6.22		4.94 (1.07)
Fluency	3.40-5.80	4.80 (0.97)	2.60-6.20		4.74 (1.31)	2.60-6.20		4.77 (1.13)
Absorption	4.75-6.50	5.30 (0.72)	2.25-6.25		5.14 (1.37)	2.25-6.50		5.21 (1.10)
Perceived importance	2.00-5.33	3.67 (1.20)	1.33-5.33		3.52 (1.65)	1.33-5.33		3.58 (1.42)

^a^FSS items were rated on a 7-point Likert scale ranging from 1 (“not at all”) to 7 (“very much”).

### Thematic Analysis

The focus group discussions elicited a wealth of insights centered around students’ views of the game’s fun and enjoyability, the degree to which they found it engaging, and its effectiveness as a rehearsal tool. Students were mostly in agreement in their perceptions of the game and persistent in their attempts to offer suggestions on how to improve the intervention. For reasons of confidentiality, all participant quotations mentioning names and the speakers themselves were deidentified. Students were then issued a unique code (eg, G2B) for identification among the study team. All participants were Singaporeans and communicated primarily in the local vernacular, Singlish, an English-based creole language; while comprised almost completely of English words, Singlish uses a grammatical structure that may be perceived to be incorrect by a nonlocal English speaker. To avoid accidental changes in meaning, quotations retained conversational Singlish quotations verbatim, such as the term “ya,” an analog of “yes,” usually used as an affirmation, and the ubiquitous particle “lah,” which is typically used at the end of a sentence that, when combined with an appropriate tone, may modify or emphasize the meaning of an entire utterance akin to the use of adverbs on a target word.

### Perceptions of Fun and Enjoyment

Participants could differentiate between the educative rehearsal components and the actual game activity, with many expressing surprise that the board game could contain elements they perceived to be fun and enjoyable. These elements were attributable to the game’s flexible and unrestrictive rule set, with notable moments of cooperation during the mid- and late stages of gameplay, where teams formed temporary alliances against particularly competent players.

Then I was surprised with the fact that, I mean, if you put all the academics aside, it really functions as like a real game and real strategies.

Student G2B

I don’t know whether the original game was supposed to be about forming alliances, but I think it’s very fun to be able to form alliances.

Student G1E

Of note, and although not surfaced during focus group discussions, the formation of alliances was often accompanied by friendly banter between parties, including those left out of said alliance.

### Perceptions of Engagement

Although students required time to familiarize themselves with the intervention, most eventually found the activity capable of engagement and sustained attention. This was attributed to the game’s interactive component, such as the manipulation of objects in the digital space. Interaction with other players, such as forming alliances against a common foe, appeared to facilitate engagement alongside the previously noted fun and enjoyability.

I think it was very cool...like, you can move the cards around, like, actual board game but it’s online.

Student G1A

...so that’s why we formed alliance against them. [Laughing]. To stop their progress across the board.

Student G1B

The use of strategy appeared to be directly facilitated by the competitive element. However, the risk of losing one’s units occasionally resulted in feelings of frustration, especially when facing two allied teams, to the point that students needed reminders on the game’s comeback mechanics.

Cause, I mean, you can’t...it’s like you have to choose someone to target, and then it’s easier to target one person than all.

Student G2C

Beyond this, students also noted that the mere notion of a play activity immediately after the rehearsal task acted as an incentive to continue partaking in the rehearsal activity. Once engaged, students appreciated a balance between the difficulty of question cards and their own skill.

...I think it also gives us an incentive to actually...remember the, like, the questions...so it makes it more fun to learn.

Student G1C

And the questions...they weren’t, like, very easy. Like, it’s not, like everyone just get correct.

Student G1A

Finally, students were willing to re-engage with the game intervention immediately after the conclusion of the focus group discussions and, thus, the study. These views were not captured as a result, but they were taken as observational findings by the study team that the intervention was acceptably engaging for use in rehearsal.

### Perceptions of the Rehearsal Tool

Most students were appreciative that the rehearsal tool provided them with immediate feedback, although some requested more detailed explanations for correct answers. Overall, students reported positive impressions of the rehearsal activity, in particular, the time limit, chance for immediate rewards, and that all students could view the questions at the same time.

Ya, but if there’s like explanations...like, a sort of like answer booklet that is quite detailed.

Student G2F

And the questions are quite relevant to what we’ve already studied. So, it’s...a better grasp of what, like, we see in the labs.

Student G1E

Now this they’re introduced more of like VR, but like, I think having, like, a board game really takes it to a different level.

Student G1D

### Student Perceptions of Flow

When discussing their inputs into the FSS, which was conducted prior to the focus group discussion, most students were able to pinpoint the time that they first noticed themselves entering a state of flow.

I think at the point where you managed to destroy your first unit...that’s the point where you really get into the game.

Student G1B

Like, the first round of questions when you got to see, like, a taste of what the questions are like. Then it, it made me quite involved really...like, that was when I was like, like, when I was in the zone.

Student G1E

Other students did not observe themselves entering a flow state and cited reasons of fatigue and the continuous rotation between rehearsal and play hindering the commencement of a flow state.

I don’t think I reached any state of flow. [Laughing]. There [was] no coffee.

Student G1F

I mean if you’re playing a game, of course you enter the flow, just like don’t see time going, but this is still like sort of studying and playing.

Student G2B

However, students who did not perceive themselves as having entered flow states also noted an altered perception of time and the suspension of fatigue until after the conclusion of the intervention, both possible indicators of flow.

Ya, I didn’t feel the one hour.

Student G2B

Just that you couldn’t...You feel fatigued but you don’t feel that it is one hour long. Ya.

Student G2D

### Student Recommendations for Improvement

Students offered a surprising number of suggestions on how the game could be improved. Suggestions included the need for automation, more complexity and opportunities to strategize during the play phase of each team’s turn, even more powerful magic cards with opportunities to use them, a faster overall game pace, and gamification of the rehearsal element through a “snatching” feature, wherein a team who is unable to answer, or incorrectly answers, a question will open that question to the other two teams for answering.

Like there’s no, there’s no like superpower card or something whereby you can be like, oh, you suddenly can ward off all the damages.

Student G2D

I think it’s a bit slow...So I think, like, you can just reduce the, the number of steps in, like, every direction. So just shorter overall.

Student G1A

Maybe if your team cannot answer the question you can ask other teams to answer.

Student G2F

I think they should automate all the, like, the placing of the units and stuff. And then sometimes you also forget to move the thing.

Student G1D

## Discussion

### Principal Findings

The creation of active student-centered learning environments that promote learning, participation, and engagement has long been a need in education [55]. To this end, the exploratory intervention in this study appears to have been successful, due to students mostly perceiving it as a fun, enjoyable activity that suitably facilitated gamified rehearsal and engagement, the latter of which appeared to occur based on the moderate degrees of flow indicated by results of the FSS. Although it had not been the original intention, the intervention, once subject to a few minor revisions based on feedback, appears suitable for a student-centered model of rehearsal due to its dynamic nature, unpredictability due to elements of luck and strategy, and means for students to exert control over the learning process [3,56,57].

While the use of strategy appeared most linked to perceptions of fun, enjoyability, and engagement, to the best of our knowledge this study appears to be the only medical student–focused digital board game intervention, if not the only such intervention, to have encountered such a finding. While numerous publications have described games requiring the use of strategy, study designs and subject population types largely varied enough that none were able to draw similar conclusions [29-40]. Notably, research in serious games commonly features validation and efficacy studies, thus minimizing the chance for such conclusions to be drawn [11,12,20-23,29-40].

However, findings from this study bear similarities to gamified rehearsal-based interventions designed to complement existing teaching. While not an explicit game like the one in this study, the TERMInator tool, developed by Seidlein and colleagues [58] to facilitate the rehearsal of medical terminologies, was noted to be an approachable tool that students were inclined to engage with, most likely due to the introduction of gamified rehearsal.

Such findings also bore similarities to results from motivational reinforcers used to enhance retention in cognitive therapies, such as task-switching in young children with cognitive control problems, such as attention deficit hyperactivity disorder [59]. While not explicitly stated, when such reinforcers comprised the introduction of game or game-like elements that required the use of strategy in overcoming tasks, motivation to continue with an otherwise unattractive task was noted to increase across both laboratory and commercialized cognitive training tools [59-61]. Although the intent of such interventions is to train, as opposed to rehearse, the similarities of the findings despite differing approaches remain notable.

Nonetheless, medical student subjects from a study examining the only other anatomy-based board game, published in 2014 by Anyanwu [40], reported similar perceptions, such as interest and enjoyment, despite the two studies’ differing theoretical foundations, approaches, and intents. The findings of Moro and colleagues [62], who created a physiology and anatomy revision game, also bore similarities to this study in that the interweaving of rehearsal and game elements likely contributed to increased motivation and engagement as well as a more positive view of the rehearsal activity.

Aside from the use of strategy, most perceptions of fun and enjoyability were linked to interactions with one’s teammate, opponents, and the play activity itself. Due to the aforementioned focus on validation studies [28-39], the authors were unable to identify any studies whose designs allowed for the direct attribution of enjoyment to any of the interpersonal relations, although overarching perceptions of fun, most likely with the actions of play, were still observed [40]. However, existing research into the conduciveness of learning environments has suggested that decreasing or disallowing both teacher-student and student-student interactions, thereby decreasing the quality of social interactions, interferes with beliefs of self-efficacy and self-concept of ability [63-65]. The apparent underreporting on whether an intervention is fun, enjoyable, or engaging is possibly linked to the presumption that it must be these things if it was to be successful. However, this paper posits that not knowing if a game-based activity is enjoyable risks misattributing efficacious results to other factors, such as the benefits of increased exposure even if an intervention is not engaging [66,67].

Instead, findings of a similar nature have been noted in gamified, as opposed to explicit game-based, interventions. Felszeghy and colleagues [68] noted that a gamified and collaborative team-based approach to histology education among dental and medical students resulted in increased motivation to learn and interest in histology, and it scored high in participant satisfaction. While they also noted that their students appeared to prefer histology learning materials in traditional mediums as opposed to reading them off of screens [68], this was not surfaced in the focus group discussions of this study, likely due to the conciseness of the question cards.

Although the intention was for the rehearsal activity to remain minimally gamified to preserve the efficacy of known rehearsal methods, students held opposing views and suggested a means for teams to “snatch” a question card and its benefits, should the drawing team be unable to answer it. While similar features, though uncommon, have previously been noted in the literature to no obvious detriment [40], such a feature is not likely to negatively impact the rehearsal activity. Presently, any one team may wait up to 2 minutes between their turns, and unexpected delays (ie, a team taking too long to make their moves) may risk onset of boredom and distract from engaging with the activity. While this was not observed in this exploratory study, it remained one of the justifications for setting a 30-second time limit to answer question cards, and such a rule should serve only to maintain psychological arousal until a team’s next turn.

Of note, engagement appeared most strongly linked with fun and enjoyability, as supported by the findings from the FSS, which indicated a moderate degree of absorption and overall flow. A possible explanation is the free-spirited and unrestrained nature of the play component, which appeared to allow students to exercise creativity when strategizing against their opponents, resulting in an intrinsically motivating desire to continue partaking in the game. An unexpected development was the formation of informal alliances between two teams against a more competent third team who, by virtue of correctly answering many question cards, was able to control significant portions of the game board with more and stronger units. Interestingly, the formation of such alliances was not always deemed negative by the third team, despite such behaviors usually being considered negative in real-world settings [69,70]. This was especially true if the third team was still able to maintain an advantage, despite two teams uniting against them.

Feelings of frustration were noted when two allied teams were able to push back a third team, but these were, to a degree, alleviated by the game’s comeback mechanics, in particular, a rule where any team who loses a life is automatically allowed to draw a magic card. Such feelings are often unavoidable in games requiring some manner of human collaboration [70], and the inclusion of such comeback mechanics based on SDT was helpful in easing frustration. Although students had refrained from mentioning this during the focus group discussions, alliances, due to their informal nature [70], were sometimes broken by teams wishing to exploit opportunities (ie, securing the star tile at the central intersection) and press advantages. Such betrayals occurred infrequently and did not appear to be negatively received by their peers, and it was decided that rules against such behaviors were unnecessary at this time.

Although the method (ie, question cards) of rehearsal was positively received by students, many students disagreed with the choice to present question cards from the entirety of their anatomy education, deviating from an identical design choice of similar games [40]. While this was deemed suitable due to all subjects having completed their anatomy modules by the time of the study, students instead preferred having the option to pick questions from card decks sorted by both topic and difficulty, such that they may opt for an easier question in exchange for a lesser reward should they not feel confident in their mastery of any such topic. This preference is likely attributable to the digital flashcard smartphone apps commonly used by students for rehearsal, such as Anki [71,72], due to their similarities, but note that there is little in the literature that indicates the superiority of one method over the other.

A contrast was noted between the perceived importance of the activity as indicated by the FSS and what was verbally spoken during the focus group discussion. Although students appeared immensely invested at the prospect of gamified rehearsal, as evidenced by the enormous amount of feedback and suggestions they provided, the mean perceived importance of the activity was rated a low value of 3.59. While the rationale for such a discrepancy could not be discerned from the available data, it is possible that the playing of games, even for rehearsal purposes, is ultimately given a lower priority for students in their second year, due to the many undertakings expected of them during their education. It is also likely, as mentioned by one student, that students were able to discern that the proper use of gamification results in the exchange of some efficiency for motivation and is often used when traditionally efficient methods no longer suffice; thus, they rated the activity accordingly.

Finally, although the intervention may readily serve as an additional means of rehearsal, the game may also serve as a capstone event, such as a course-wide gaming challenge, to further reinforce what students have learned during their education. Competitive learning tools, when well used, have been known to improve motivation and satisfaction in blended learning environments [73,74]. Coupled with the higher-than-average levels of competitiveness observed in medical students [75], this appears to be a prospective avenue warranting further investigation.

### Limitations

Due to the exploratory nature of this study, the authors were neither able to predict findings, such as the formation of informal alliances and the unusually low levels of importance given to the activity, nor generate hypotheses, a priori or otherwise, regarding their nature. Although the gamification of rehearsal appears well received and helpful, the exploratory nature of this study serves instead as a starting point from which further research, both qualitative and quantitative, may commence to identify further means of increasing efficiency when compared to traditional methods. The small sample size and broad distribution of data have also limited the interpretation of findings from the FSS. Due to the focus on confirming the feasibility of the intervention, the game’s educational impact may only be determined in a follow-up study once necessary improvements have been made. An element of self-selection bias, in favor of students with existing interests toward playing board games, may have been present due to the nature of the study and voluntary nature of recruitment.

### Conclusions

This study demonstrated the evidence to support the use of SDT as an appropriate theoretical base to develop game rules and features that facilitate a high degree of intrinsic motivation in a digital educational intervention. The new digital board game of anatomy examined in this study appears to hold its promise as an intervention for rehearsal and practice of learned material in a fun and enjoyable environment.

## Abbreviations

FSS: Flow Short Scale
MBBS: Bachelor of Medicine, Bachelor of Surgery
SDT: self-determination theory

## References

[1] Smolle J (2016). Complexity and medical education. Saf Health.

[2] Woodruff JN (2019). Accounting for complexity in medical education: A model of adaptive behaviour in medicine. Med Educ.

[3] Ang ET, Chan JM, Gopal V, Li Shia N (2018). Gamifying anatomy education. Clin Anat.

[4] Ramnanan C, Pound L (2017). Advances in medical education and practice: Student perceptions of the flipped classroom. Adv Med Educ Pract.

[5] Chen F, Lui AM, Martinelli SM (2017). A systematic review of the effectiveness of flipped classrooms in medical education. Med Educ.

[6] Poeppelman R, Liebert C, Vegas D, Germann C, Volerman A (2016). A narrative review and novel framework for application of team-based learning in graduate medical education. J Grad Med Educ.

[7] Baddeley A (1997). Human Memory: Theory and Practice. Revised edition.

[8] Schöne C, Tandler SS, Stiensmeier-Pelster J (2015). Contingent self-esteem and vulnerability to depression: Academic contingent self-esteem predicts depressive symptoms in students. Front Psychol.

[9] Woods NN, Brooks LR, Norman GR (2007). It all make sense: Biomedical knowledge, causal connections and memory in the novice diagnostician. Adv Health Sci Educ Theory Pract.

[10] Custers EJFM (2010). Long-term retention of basic science knowledge: A review study. Adv Health Sci Educ Theory Pract.

[11] Gentry SV, Gauthier A, L'Estrade Ehrstrom B, Wortley D, Lilienthal A, Tudor Car L, Dauwels-Okutsu S, Nikolaou CK, Zary N, Campbell J, Car J (2019). Serious gaming and gamification education in health professions: Systematic review. J Med Internet Res.

[12] Bigdeli S, Kaufman D (2017). Digital games in medical education: Key terms, concepts, and definitions. Med J Islam Repub Iran.

[13] Heimo OI, Harviainen JT, Kimppa KK, Mäkilä T (2016). Virtual to virtuous money: A virtue ethics perspective on video game business logic. J Bus Ethics.

[14] Alvarez J, Djaouti D (2010). Introduction au Serious Game.

[15] Gorbanev I, Agudelo-Londoño S, González RA, Cortes A, Pomares A, Delgadillo V, Yepes FJ, Muñoz Ó (2018). A systematic review of serious games in medical education: Quality of evidence and pedagogical strategy. Med Educ Online.

[16] Stieglitz S, Lattemann C, Susanne S, Zarnekow R, Brockmann T (2017). Gamification: Using Game Elements in Serious Contexts.

[17] Dankbaar MEW, Richters O, Kalkman CJ, Prins G, Ten Cate OTJ, van Merrienboer JJG, Schuit SCE (2017). Comparative effectiveness of a serious game and an e-module to support patient safety knowledge and awareness. BMC Med Educ.

[18] See C, Chan LK, Pawlina W (2020). Gamification in anatomy education. Teaching Anatomy: A Practical Guide.

[19] Michailidis L, Balaguer-Ballester E, He X (2018). Flow and immersion in video games: The aftermath of a conceptual challenge. Front Psychol.

[20] IJgosse W, van Goor H, Rosman C, Luursema J (2020). Construct validity of a serious game for laparoscopic skills training: Validation study. JMIR Serious Games.

[21] Jalink MB, Heineman E, Pierie JPEN, ten Cate Hoedemaker HO (2015). The effect of a preoperative warm-up with a custom-made Nintendo video game on the performance of laparoscopic surgeons. Surg Endosc.

[22] Boada I, Rodriguez Benitez A, Thió-Henestrosa S, Soler J (2020). A serious game on the first-aid procedure in choking scenarios: Design and evaluation study. JMIR Serious Games.

[23] Alyami H, Alawami M, Lyndon M, Alyami M, Coomarasamy C, Henning M, Hill A, Sundram F (2019). Impact of using a 3D visual metaphor serious game to teach history-taking content to medical students: Longitudinal mixed methods pilot study. JMIR Serious Games.

[24] Epstein DS, Zemski A, Enticott J, Barton C (2021). Tabletop board game elements and gamification interventions for health behavior change: Realist review and proposal of a game design framework. JMIR Serious Games.

[25] Flores RL, Silverio R, Feria R, Cariaga AA, Tlili A, Chang M (2019). Motivational factors through learning analytics in digital game-based learning. Data Analytics Approaches in Educational Games and Gamification Systems.

[26] Snodgrass Rangel V, Bell ER, Monroy C, Whitaker JR (2015). Toward a new approach to the evaluation of a digital curriculum using learning analytics. J Res Technol Educ.

[27] Bayeck RY (2020). Examining board gameplay and learning: A multidisciplinary review of recent research. Simul Gaming.

[28] Xu Y, Barba E, Radu I, Gandy M, Macintyre B (2011). Chores are fun: Understanding social play in board games for digital tabletop game design. Proceedings of the 2011 DiGRA International Conference: Think Design Play.

[29] Gilliam M, Hill BJ, Jaworski E, Sparrow A, Jones IB, Jagoda P (2019). Increasing anti-tobacco industry attitudes among youth: A pilot study of a multiplayer educational board game. Games Health J.

[30] Viggiano A, Viggiano E, Di Costanzo A, Viggiano A, Andreozzi E, Romano V, Rianna I, Vicidomini C, Gargano G, Incarnato L, Fevola C, Volta P, Tolomeo C, Scianni G, Santangelo C, Battista R, Monda M, Viggiano A, De Luca B, Amaro S (2015). Kaledo, a board game for nutrition education of children and adolescents at school: Cluster randomized controlled trial of healthy lifestyle promotion. Eur J Pediatr.

[31] Viggiano E, Viggiano A, Di Costanzo A, Viggiano A, Viggiano A, Andreozzi E, Romano V, Vicidomini C, Di Tuoro D, Gargano G, Incarnato L, Fevola C, Volta P, Tolomeo C, Scianni G, Santangelo C, Apicella M, Battista R, Raia M, Valentino I, Palumbo M, Messina G, Messina A, Monda M, De Luca B, Amaro S (2018). Healthy lifestyle promotion in primary schools through the board game Kaledo: A pilot cluster randomized trial. Eur J Pediatr.

[32] Wanyama J, Castelnuovo B, Robertson G, Newell K, Sempa J, Kambugu A, Manabe YC, Colebunders R (2012). A randomized controlled trial to evaluate the effectiveness of a board game on patients' knowledge uptake of HIV and sexually transmitted diseases at the Infectious Diseases Institute, Kampala, Uganda. J Acquir Immune Defic Syndr.

[33] Maclachlan M, Chimombo M, Mpemba N (1997). AIDS education for youth through active learning: A school-based approach from Malawi. Int J Educ Dev.

[34] Lennon JL, Coombs DW (2007). The utility of a board game for dengue haemorrhagic fever health education. Health Educ.

[35] Akogun OB (1992). The effect of selected health education schemes on knowledge and attitude of the Kanuri towards certain parasitic diseases. J R Soc Health.

[36] Van Scoy LJ, Green MJ, Reading JM, Scott AM, Chuang CH, Levi BH (2017). Can playing an end-of-life conversation game motivate people to engage in advance care planning?. Am J Hosp Palliat Care.

[37] Sen M, Uzuner A, Akman M, Bahadir AT, Borekci NO, Viggiano E (2018). Examination of a board game approach to children's involvement in family-based weight management vs traditional family-based behavioral counseling in primary care. Eur J Pediatr.

[38] Crawford P, Wiltz S (2015). Participation in the Journey to Life Conversation Map improves control of hypertension, diabetes, and hypercholesterolemia. J Am Board Fam Med.

[39] van der Stege HA, van Staa A, Hilberink SR, Visser AP (2010). Using the new board game SeCZ TaLK to stimulate the communication on sexual health for adolescents with chronic conditions. Patient Educ Couns.

[40] Anyanwu EG (2014). Anatomy adventure: A board game for enhancing understanding of anatomy. Anat Sci Educ.

[41] Robson K, Plangger K, Kietzmann JH, McCarthy I, Pitt L (2015). Is it all a game? Understanding the principles of gamification. Bus Horiz.

[42] Limmanee A (2020). Review of esports and video game research with analysis of "Among Us" game casting. Int J Ind Educ Technol.

[43] Earle E (2021). An impostor “among us”: Teaching group development and cohesion online. Commun Teach.

[44] Barab SA, Gresalfi M, Dodge T, Ingram-Goble A (2010). Narratizing disciplines and disciplinizing narratives: Games as 21st century curriculum. Int J Gaming Comput Mediat Simul.

[45] Thomas TH, Sivakumar V, Babichenko D, Grieve VLB, Klem ML (2020). Mapping behavioral health serious game interventions for adults with chronic illness: Scoping review. JMIR Serious Games.

[46] Greenhalgh T, Plsek P, Wilson T, Fraser S, Holt T (2010). Response to 'The appropriation of complexity theory in health care'. J Health Serv Res Policy.

[47] Shiell A, Hawe P, Gold L (2008). Complex interventions or complex systems? Implications for health economic evaluation. BMJ.

[48] DeSmet A, Van Ryckeghem D, Compernolle S, Baranowski T, Thompson D, Crombez G, Poels K, Van Lippevelde W, Bastiaensens S, Van Cleemput K, Vandebosch H, De Bourdeaudhuij I (2014). A meta-analysis of serious digital games for healthy lifestyle promotion. Prev Med.

[49] Johnson D, Deterding S, Kuhn K, Staneva A, Stoyanov S, Hides L (2016). Gamification for health and wellbeing: A systematic review of the literature. Internet Interv.

[50] Deci EL, Ryan RM (2000). The "what" and "why" of goal pursuits: Human needs and the self-determination of behavior. Psychol Inq.

[51] Deci EL, Ryan RM (1985). Intrinsic Motivation and Self-Determination in Human Behavior.

[52] Kyriazos TA, Stalikas A, Prassa K, Galanakis M, Flora K, Chatzilia V (2018). The Flow Short Scale (FSS) dimensionality and what MIMIC shows on heterogeneity and invariance. Psychology.

[53] Rheinberg F, Vollmeyer R, Engeser S, Stiensmeier-Pelster J, Rheinberg G (2003). Assessment of flow experiences. Diagnosis of Motivation and Self-Concept. Tests and Trends N.F. Band 2 [Book in German].

[54] Braun V, Clarke V (2006). Using thematic analysis in psychology. Qual Res Psychol.

[55] Blakely G, Skirton H, Cooper S, Allum P, Nelmes P (2009). Educational gaming in the health sciences: Systematic review. J Adv Nurs.

[56] Oblinger DG (2004). The next generation of educational engagement. J Interact Media Educ.

[57] Jones L (2007). The Student-Centered Classroom.

[58] Seidlein A, Bettin H, Franikowski P, Salloch S (2020). Gamified e-learning in medical terminology: The TERMInator tool. BMC Med Educ.

[59] Bioulac S, Lallemand S, Fabrigoule C, Thoumy A, Philip P, Bouvard MP (2014). Video game performances are preserved in ADHD children compared with controls. J Atten Disord.

[60] Klingberg T, Forssberg H, Westerberg H (2002). Training of working memory in children with ADHD. J Clin Exp Neuropsychol.

[61] Prins PJ, Brink ET, Dovis S, Ponsioen A, Geurts HM, de Vries M, van der Oord S (2013). "Braingame Brian": Toward an executive function training program with game elements for children with ADHD and cognitive control problems. Games Health J.

[62] Moro C, Phelps C, Stromberga Z (2020). Utilizing serious games for physiology and anatomy learning and revision. Adv Physiol Educ.

[63] Eccles JS, Midgley C, Wigfield A, Buchanan CM, Reuman D, Flanagan C, Mac Iver D (1993). Development during adolescence: The impact of stage-environment fit on young adolescents' experiences in schools and in families. Am Psychol.

[64] Ames R, Ames C (1984). Research on Motivation in Education: Goals and Cognitions.

[65] Wigfield A, Eccles J, Fredricks J, Simpkins S, Roeser R, Schiefele U, Lerner R (2015). Development of achievement motivation and engagement. Handbook of Child Psychology and Developmental Science. 7th edition.

[66] McCambridge J, de Bruin M, Witton J (2012). The effects of demand characteristics on research participant behaviours in non-laboratory settings: A systematic review. PLoS One.

[67] Greenberg BG, Abul-Ela AA, Simmons WR, Horvitz DG (1969). The unrelated question randomized response model: Theoretical framework. J Am Stat Assoc.

[68] Felszeghy S, Pasonen-Seppänen S, Koskela A, Nieminen P, Härkönen K, Paldanius KMA, Gabbouj S, Ketola K, Hiltunen M, Lundin M, Haapaniemi T, Sointu E, Bauman EB, Gilbert GE, Morton D, Mahonen A (2019). Using online game-based platforms to improve student performance and engagement in histology teaching. BMC Med Educ.

[69] Tortoriello GK, Hart W, Breeden CJ (2020). Of malevolence and morality: Psychopathy dimensions are conducive to helping in highly-distressing moral dilemmas. Pers Individ Dif.

[70] Zagal JP, Rick J, Hsi I (2016). Collaborative games: Lessons learned from board games. Simul Gaming.

[71] Yeh DD, Park YS (2015). Improving learning efficiency of factual knowledge in medical education. J Surg Educ.

[72] Pumilia C, Lessans S, Harris D (2020). An evidence-based guide for medical students: How to optimize the use of expanded-retrieval platforms. Cureus.

[73] Hwang G, Wu P, Chen C (2012). An online game approach for improving students’ learning performance in web-based problem-solving activities. Comput Educ.

[74] Verdú E, Regueras LM, Verdú MJ, Leal JP, de Castro JP, Queirós R (2012). A distributed system for learning programming on-line. Comput Educ.

[75] Lempp H, Seale C (2004). The hidden curriculum in undergraduate medical education: Qualitative study of medical students' perceptions of teaching. BMJ.

